# Diversity of *Francisella tularensis* Subsp. *holarctica* Lineages, China

**DOI:** 10.3201/eid2007.130931

**Published:** 2014-07

**Authors:** Yanhua Wang, Yao Peng, Rong Hai, Lianxu Xia, Hang Li, Zhikai Zhang, Hong Cai, Ying Liang, Xiaona Shen, Dongzheng Yu, Dawn Birdsell, David M. Wagner, Paul Keim

**Affiliations:** National Institute for Communicable Disease Control and Prevention;; State Key Laboratory for Infectious Disease Prevention and Control;; Collaborative Innovation Center for Diagnosis and Treatment of Infectious Diseases, Beijing, China (Y. Wang, Y. Peng, R. Hai, L. Xia, Z. Zhang, H. Cai, Y. Liang, X. Shen, D. Yu);; Beijing Genomics Institute–Shenzhen, Shenzhen, China (H. Li);; Northern Arizona University, Flagstaff, Arizona, USA (D. Birdsell, D.M. Wagner, P. Keim)

**Keywords:** Francisella tularensis subsp. holarctica, phylogeography, SNP, canSNP, China, bacteria, tularemia, lineage

## Abstract

We analyzed 10 isolates of *Francisella tularensis* subspecies *holarctica* from China and assigned them to known clades by using canonical single-nucleotide polymorphisms. We found 4 diverse subtypes, including 3 from the most basal lineage, biovar *japonica*. This result indicates unprecedented levels of diversity from a single region and suggests new models for emergence.

Tularemia is a disease caused by distinct subspecies and phylogenetic groups within the bacterial species *Francisella tularensis* (*1**,**2*). These groups exhibit distinct phylogeographic patterns; *F. tularensis* subsp. *tularensis* (type A) is restricted to North America, whereas *F. tularensis* subsp. *holarctica* (type B) is found throughout many parts of the Northern Hemisphere (*3*) and has been reported recently in Tasmania (*4*). Both subspecies exhibit highly clonal population structures, as determined by phylogenetic analysis using data from multilocus variable number tandem repeat analysis, single-nucleotide polymorphisms (SNPs), and indels (*5**–**7*). The wide geographic distribution and low diversity of *F. tularensis* subsp. *holarctica* isolates have been used to argue that this clade is recently emerged and highly fit (*3*), but the geographic origin of its emergence has not been determined.

*F. tularensis* subsp. *holarctica* has been further subdivided by whole-genome sequencing and canonical SNP (canSNP) genotyping into multiple clades (*7*) (Figure 1). The most basal clade consists of strains assigned to the biovar *japonica*; this biovar had previously only been reported from Japan (*8*), but a recent report suggests that it may be found in Turkey (*9*). The next derived clade (B.2/3) has been described only from 2 isolates from California, USA (*7*). Isolates from these 2 most basal clades are rare, and apparently geographically restricted, but still provide insights into the origin of *F. tularensis* subsp. *holarctica*. The global expansion of the more derived clades is extensive, and closely related isolates are common and widely distributed. The source for emergence of the main type B has been proposed for either North America or Scandinavia, on the basis of the presence of the OSU18 clade isolates in both locations (*6**,**7*). However, a sampling bias toward both of these geographic regions has left *F. tularensis* subsp. *holarctica* diversity in much of the rest of the world poorly understood. We analyzed 10 isolates of *F. tularensis* subsp. *holarctica* from China (*10*) to determine their placement within the current global phylogeographic framework of this pathogen.

**Figure 1:**
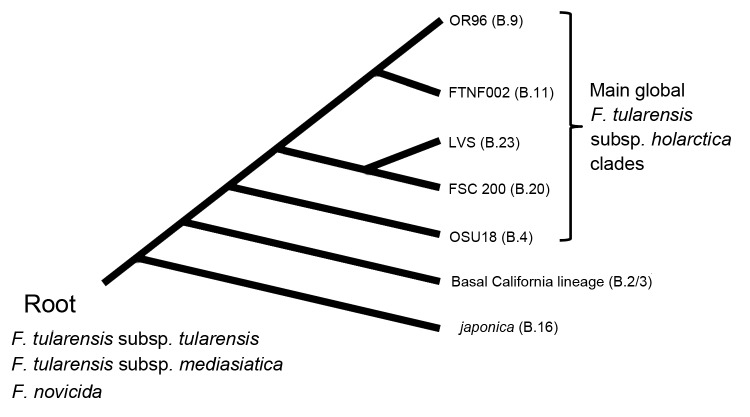
Evolutionary development of *Francisella tularensis* subsp. *holarctica*. Previous studies (*6**,**7**,**11*) defined the major lineages of this subspecies on the bases of whole-genome sequences and single-nucleotide polymorphism analysis. The other *F. tularensis* subspecies and closely-related *Francisella* species are shown at the root.

## The Study

The *F. tularensis* subsp. *holarctica* isolates we analyzed were collected over a long period but have been preserved by lyophilization and have been verified every 5 years since they were isolated (Table 1, Appendix). We assigned these isolates into previously defined (*6**,**7*) phylogenetic clades and conducted a phylogeographic analysis by using a panel of 12 canSNPs specific for *F. tularensis* subspecies or clades within *F. tularensis* subsp. *holarctica* (Table 2); these canSNPs were obtained from previous reports (*6**,**7*). The canSNP analysis was PCR based and performed as described (*7*). Table 1, Appendix, lists the derived or ancestral allele status for these isolates and for 13 control isolates. These data facilitated the assignment of the 10 *F. tularensis* subsp. *holarctica* isolates to major phylogenetic subgroups previously identified within this subspecies (*6**,**7*).

**Table 1:** *Francisella tularensis* isolates used in study of lineages in China*

Strain ID	Year of isolation	Region and country	Subspecies or species	Source	F.3 910179†	B.2 5162‡	B.3 470841‡	B.4 823672‡	B.5 1853655‡	B.6 713647‡	B.12 109781‡	B.16 608245§	B.17 1743207§	B.19 1373999§	B.20 1396082, 1789417§	B.23 253120§	Major subgroup¶	Subgroup#
410108	1962	Tibet, China	subsp. *holarctica*	Human	G	C	G	A	C	G	A	T	C	C	T	C	BV	B.16
410109	1962	Tibet, China	subsp. *holarctica*	Human	G	C	G	A	C	G	A	T	C	C	T	C	BV	B.16
410111	1964	Tibet, China	subsp. *holarctica*	Tick (*D. everestianus*)	G	C	G	A	C	G	A	T	C	C	T	C	BV	B.16
FSC022**	1950	Japan	subsp. *holarctica*	Human	G	C	G	A	C	G	A	T	C	C	T	C	BV	B.16
410107	Pre-1992	Heilongjiang, China	subsp. *holarctica*	Hare	G	A	A	A	T	G	T	G	C	A	C	C	BI	B.20
410116	1983	Xinjiang, China	subsp. *holarctica*	Tick	G	A	A	A	T	G	T	G	C	A	C	C	BI	B.20
410117	Pre-1992	Xinjiang, China	subsp. *holarctica*	Tick	G	A	A	A	T	G	T	G	C	A	C	C	BI	B.20
FSC200**	1998	Sweden	subsp. *holarctica*	Human	G	A	A	A	T	G	T	G	C	A	C	C	BI	B.20
RC503/ FSC257**	1949	Russia	subsp. *holarctica*	Tick (*D. pictus*)	G	A	A	A	T	G	T	G	C	A	T	A	BIII	B.23
LVS**	UNK	Russia	subsp. *holarctica*	UNK	G	A	A	A	T	G	T	G	C	A	T	A	BIII	B.23
410112	1971	Tibet, China	subsp. *holarctica*	Tick (*D. everestianus*)	G	A	A	T	C	G	A	G	A	C	T	C	BII	B.4
410113	1964	Tibet, China	subsp. *holarctica*	Tick (*D. everestianus*)	G	A	A	T	C	G	A	G	A	C	T	C	BII	B.4
920607	Pre-1992	Tibet, China	subsp. *holarctica*	UNK	G	A	A	T	C	G	A	G	A	C	T	C	BII	B.4
OSU18**	1978	Oklahoma, United States	subsp. *holarctica*	Beaver	G	A	A	T	C	G	A	G	A	C	T	C	BII	B.4
MI00-1730**	2000	Michigan, United States	subsp. *holarctica*	Human	G	A	A	T	C	G	A	G	A	C	T	C	BII	B.4
410105	1962	Tibet, China	subsp. *holarctica*	Human	G	A	A	A	T	A	A	G	C	C	T	C	BIV	B.6
FTNF002-00**	UNK	France	subsp. *holarctica*	UNK	G	A	A	A	T	A	A	G	C	C	T	C	BIV	B.6
URFT1**	1997	France	subsp. *holarctica*	Human	G	A	A	A	T	A	A	G	C	C	T	C	BIV	B.6
F92**	2004	Germany	subsp. *holarctica*	Marmoset	G	A	A	A	T	A	A	G	C	C	T	C	BIV	B.6
OR96-0246**	1996	Oregon, United States	subsp. *holarctica*	UNK	G	A	A	A	T	A	A	G	C	C	T	C	BIV	B.6
Schu S4**	1941	Ohio, United States	subsp. *tularensis*	Human	A	C	G	A	C	G	A	G	C	C	T	C	NA	NA
FSC147**	1965	Kazakhstan	subsp. *mediasiatica*	Gerbil	A	C	G	A	C	G	A	G	C	C	T	C	NA	NA
U112**	1950	Utah, United States	*F. novicida*	Water	A	C	G	A	C	G	A	G	C	C	T	C	NA	NA

*Clade designations are underlined. Gray shading indicates derived SNPs; other SNPs are ancestral. ID, Identification; UNK, unknown; NA, not applicable; SNP, single-nucleotide polymorphism. †SNP from (*10*). SNP position based on the reference Schu S4 (NC_006570)   ‡Canonical SNP from (*7*). SNP position based on the reference Schu S4 (NC_006570). §Canonical SNP from (*6*), SNP position based on the reference Schu S4 (NC_006570). ¶Major subgroup based on (*6*). #Subgroup based on (*6**,**7*). **Reference strain genome published in the National Center for Biotechnology Information (www.ncbi.nlm.nih.gov/genome/genomes/511).

**Table 2:** Primers used in sequencing to obtain canonical SNP loci for *Francisella tularensis* subsp. *holarctica* isolates*

SNP	SCHU S4 SNP position†	SNP state (D/A)‡	Primer sequence, 5′ → 3′	Annealing temperature, °C§
F.3	910179	G/A	F: GCTGTATCATCATTTAATAAACTGCTG	55
			R: TTGGGAAGCTTGTATCATGGCACT	
B.2	5162	A/C	F: TTAGTCTATGAGCAGCCAG	50
			R: TAATATCACCAAGGTAGCC	
B.3	470841	A/G	F: ACGCTAGGTGTCTTGGT	50
			R: CTATATCCGCTCAACAT	
B.4	823672	T/A	F: TAGACGCACTGGATTTAGGT	53.5
			R: AACCATCACGCCACCATAAG	
B.5	1853655	T/C	F: TGGATCAAACAACCGT	50
			R: TCTCAAGAGCTGGTGC	
B.6	713647	A/G	F: AGTAGTGGTAGCGAGGC	53.5
			R: TACCGTTAGCCCAACAG	
B.12	109781	T/A	F: TACTGCCCAACATAGAG	55
			R: ATCGTGATAAGGCTGGA	
B.16	608245	T/G	F: ATGCTAGCAAATTACCATCAAAAG	57
			R: AACTCTTCTCGCCATCAACTTCTAT	
B.17	1743207	A/C	F: CCAAGAGCTAAATTAGCTTCAA	53.5
			R: TGACCAAGAAGGTAGAGGTATTGGTT	
B.19	1373999	A/C	F: TTGCTACTGATGGTTTAACT	57
			R: CAATACGTCACTTATGCAGTGAT	
B.20	1396082, 1789417	C/T	F: ATGGGTCGGACTATCACATC	56
			R: ATTATTGTTAAACGGCATCG	
B.23	253120	A/C	F: GGCAACAGCAGATTCGTGAG	56
			R: TGAAAGCAGGTTTAGAAGGACAG	

*SNP, single-nucleotide polymorphism; D, derived; A, ancestral; F, forward; R, reverse. †SNP position based on the reference isolate Schu S4 (NC_006570). ‡Top strand orientation of SCHU S4. §Sequencing conditions as described in (10).

**Figure 2:**
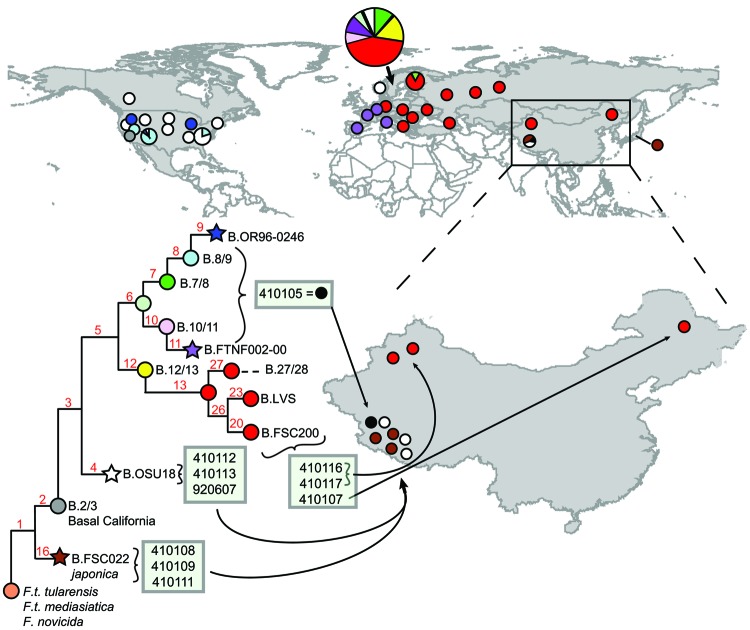
Phylogeography of *Francisella tularensis* (*F.t.*) subsp. *holarctica.* The global distribution of different clades (indicated by colored stars, circles, and circle sections) and their phylogenetic relationships (tree) are shown as described (*6**,**7**,**11*). Stars indicate sequenced reference strains. The phylogenetic positions of the 10 isolates from China (boxes on tree) and their sites of isolation (circles within China) are indicated. The exact lineage of strain 410105 (black circle) was not determined.

The isolates were of wide phylogenetic diversity for isolates from a single country. The 10 isolates we analyzed were assigned to 4 distinct phylogenetic clades: 3 were assigned to the basal *japonica* clade (B.16), 3 to the OSU18 clade (B.4), 3 to the FSC200 clade (B.20), and 1 to clade B.6 (Figure 2; Table 1, Appendix). Two of these clades are very basal (B.16 and B.4; Figure 1), whereas the other 2 are relatively derived (B.6 and B.20). Regardless, these results demonstrate the presence of multiple distinct *F. tularensis* subsp. *holarctica* lineages in China. Within China, isolates from the Tibetan plateau in the areas bordering Nepal, Bhutan, India, and central Asia were particularly diverse; all 7 strains assigned to clades B.4, B.6, and B.16 were from this region.

The substantial diversity of *F. tularensis* subsp. *holarctica* from the Tibetan region provides evidence for an Asian ancestral focus of this subspecies. With the exception of the rare B.2/3 California group, all major basal lineages were represented in this small sample from this region. The center of diversity rationale would suggest that *F. tularensis* subsp. *holarctica* diversified in Asia and then spread outward to the rest of the world. The presence of representatives of the basal *japonica* and OSU18 lineages further argues for ancestral populations in this region. In contrast, the derived and terminal position of the B.20 (FSC200) isolates in northern China suggests that this lineage was introduced to this region from other regions—perhaps Europe, in which B.20 is found (*6*)—after ancestral strains dispersed to other regions from Asia and diversified in these new locations. The analyses used in this current study show that the B.4 (OSU18) isolates from China are indistinguishable from B.4 isolates from North America or Europe and could represent an ancestral population or a reintroduction after global dissemination.

Although strong evidence shows that *F. tularensis* subsp. *holarctica* is a highly fit and recently emerged clone (*3*), we know little about the basis for its great fitness. It is possible that certain, as yet unidentified, adaptive features developed that led to an increase in its fitness. Alternatively, a stochastic event may have led to the emergence and subsequently circumpolar expansion of this subspecies. However, our understanding of the ecology of *F. tularensis* subsp. *holarctica* is severely limited, so the dispersal mechanisms that led to its wide geographic distribution have yet to be identified.

## Conclusions

Wide diversity in *F. tularensis* subsp. *holarctica* strains, including basal lineages, has been observed in China and underscores a lack of phylogeographic knowledge of this subspecies. Previous arguments (*1*) about the emergence of this highly fit subspecies have been based on highly biased sampling of strains in North America, Europe, and Japan. Our data suggest a broader distribution in Asia of the *japonica* clade (B.16) in particular. The OSU18 clade (B.4) also appears to have a broader distribution in Asia than has been observed from both North America and Europe. These clades are thought to be basal to the highly fit clonal expansion on these continents. Sampling of additional regions in Asia and characterization of those isolates would greatly advance the literature on the phylogeography of *F. tularensis* subsp. *holarctica*.
